# Extraction, Analysis, and Antioxidant Activity Evaluation of Phenolic Compounds in Different Italian Extra-Virgin Olive Oils

**DOI:** 10.3390/molecules23123249

**Published:** 2018-12-08

**Authors:** Chiara Fanali, Susanna Della Posta, Alessandra Vilmercati, Laura Dugo, Marina Russo, Tommasangelo Petitti, Luigi Mondello, Laura de Gara

**Affiliations:** 1 Unit of Food Science and Nutrition, Department of Medicine, Università Campus Bio-Medico di Roma, via Álvaro del Portillo 21, 00128 Rome, Italy; s.dellaposta@unicampus.it (S.D.P.); a.vilmercati@unicampus.it (A.V.); l.dugo@unicampus.it (L.D.); marina.russo@unicampus.it (M.R.); t.petitti@unicampus.it (T.P.); lmondello@unime.it (L.M.); l.degara@unicampus.it (L.d.G.); 2 Dipartimento di “Scienze Chimiche, Biologiche, Farmaceutiche ed Ambientali”, University of Messina-Polo Annunziata, Viale Annunziata, 98168 Messina, Italy; 3 Chromaleont S.r.L., c/o Dipartimento di “Scienze Chimiche, Biologiche, Farmaceutiche ed Ambientali”, University of Messina-Polo Annunziata, Viale Annunziata, 98168 Messina, Italy

**Keywords:** extra-virgin olive oil, protected designated origin, phenolic compounds, HPLC-PDA/MS, antioxidant activity

## Abstract

The analysis of phenolic compounds in extra virgin olive oils was carried out by high-performance liquid chromatography utilizing photodiode array and mass spectrometry detectors. The chromatographic profile of thirty samples from four Italian Regions highlighted the presence of secoiridoids, phenolic alcohols, flavonoids, and phenolic acid classes. A similar qualitative profile was observed with some differences in peak area and fifteen compounds were tentatively identified. Quantitative analysis was performed by UV detection considering eight standard phenolic compounds. The chromatographic method, after optimization, was validated studying some parameters, e.g., intra-day and inter-day retention time precision, limit of detection, limit of quantification, and linearity. Recovery of the method was performed achieving good results (10 and 50 g·g^−1^ with recovery of 72.9–92.1% (*w*/*w*) and 79.1–102.8% (*w*/*w*), respectively). In all samples secoiridoids were the main compounds ranging from 85 to more than 99% (*w*/*w*) of the total concentration of detected phenolic compounds while phenolic acids accounted for the lowest percentage (0.1–0.6%, *w*/*w*). Finally, total concentration of phenolic compounds and antioxidant activity were determined with different chemical assays. A good and significant correlation among total phenolic compound concentration and antioxidant activity was observed. A significant different phenolic compound concentration and antioxidant activity was determined between samples from Puglia and Sicily. This was studied performing statistical analysis by one-way analysis of variance (ANOVA) followed by Bonferroni post-hoc test.

## 1. Introduction

Virgin olive oil (VOO) is the product obtained from the fruit of the olive tree (*Olea europea* L.) solely by mechanical or other physical means such as washing, decantation, centrifugation, or filtration. Extra virgin olive oils (EVOOs) are classified as VOOs having a free acidity, in terms of oleic acid, lower than 0.8 g per 100 g [1]. Moreover, EVOOs whose production steps all take place in that particular region can be marked as Protected Designation of Origin (PDO). Those products present exclusive characteristics of quality and originality as a result of a particular geographical environment with its inherent natural and human factors [2].

PDO EVOOs have an added value both in quality and market price, therefore, the analysis of olive oil components that might be exploited for the discrimination on a geographical basis could be useful in the prevention of frauds and false declarations. Some techniques have been applied to pursue this aim, such as the analysis of triglyceride profiles through matrix-assisted laser desorption ionization time-of-flight (MALDI-TOF/MS), the analysis of fatty acids through gas chromatography coupled to mass spectrometry (GC-MS), and near-infrared spectroscopy (NIRS) in combination with chemometrics [3].

In addition, minor compounds such as polyphenols have been tested as marker to cluster EVOOs on the basis of their geographical origins. Ouni et al. [4] developed a liquid chromatography coupled with mass spectrometry using the electrospray ionization interface and time of flight detector (LC-ESI-TOF-MS) method for the characterization and quantification of phenolic compounds of EVOOs. They reported no qualitative, but significant quantitative, differences in a wide number of phenolic compounds in samples from different geographical regions.

Phenolic compounds are one of the most relevant classes of molecules in the unsaponifiable fraction of olive oil. Those compounds are of great interest because of their antioxidant activity, which results in several beneficial effects on human health. A wide range of polyphenols has been identified in VOO. Among them, phenolic alcohols (e.g., hydroxytyrosol, tyrosol) and their secoiridoid derivatives linked to the aldehydic and dialdehydic forms of elenolic acid (such as oleuropein, the dialdehydic form of decarboxymethylelenolic acid linked to hydroxytyrosol (3,4-DHPEA-EDA), an isomer of oleuropein aglycone (3,4-DHPEA-EA), among others) are the main ones. Flavonoids (e.g., luteolin and apigenin), phenolic acids (e.g., vanillic and p-coumaric acid) and lignans (e.g., pinoresinol) are present as minor compounds [5,6,7].

As phenolic compounds have been found only at trace levels in the oil matrix, they have to be extracted, cleaned up and concentrated to perform an accurate analytical separation and detection. Hence, some sample preparation methods have been evaluated, e.g., liquid–liquid extraction (LLE) [8,9,10,11,12,13] and solid phase extraction (SPE) [14,15] resulting the most effective. Applying LLE to the treatment of these compounds, the extraction solvent commonly employed is methanol or methanol/water mixtures (H_2_O < 40%, *v*/*v*) [16,17]. Recently LLE and SPE were applied to the pre-treatment of oil samples and the results compared [8]; no remarkable differences of the extraction efficiency of the analyzed phenolic compounds between the two methods were observed.

According to literature the most common analytical techniques used for the separation and determination of phenolic compounds in olive oil are, so far, high-performance liquid chromatography (HPLC) coupled with ultraviolet or diode array detection (PDA), electrochemical detection, mass spectrometry (MS), gas chromatography coupled to MS, capillary electrophoresis (CE) with UV or MS, nuclear magnetic resonance spectroscopy and infrared spectroscopy [8,13,17,18,19,20].

Some published papers have reported the total phenolic content in olive oils applying a spectrophotometric method (Folin-Ciocalteau) [21,22,23], while anticancer properties of separated fractions of EVOO lignans and secoiridoids were in vitro studied [19] and sample antioxidant activity determined by using different tests [24,25]. Studies concerning the antioxidant activity, describe that the natural antioxidants of EVOO with the highest antioxidant efficacy are 3,4-DHPEA and secoiridoids such as 3,4-DHPEA-EDA and 3,4-DHPEA-EA, that in their molecular structure contain 3,4-DHPEA. Samples antioxidant activity depends on the relative concentrations of these phenols in EVOOs [5].

To the best of our knowledge, some papers have been published about the phenolic compounds analysis and antioxidant activity of Italian monovarietal, blend, and PDO EVOOs. Published papers report the phenolic compounds composition of PDO oils from different Italian geographical areas with the aims to determine their concentration and compare it with that one’s of oils produced in a different way [26,27,28,29,30] or to trace the origin of EVOOs based on their phenolic compounds profile using a chemometric approach [31]. Aim of the present work was the qualitative and quantitative determination of polyphenols in samples of EVOOs produced in different Italian regions evaluating the respective fingerprinting obtained by chromatographic analyses to verify, if present, their differences.

An analytical method, for the determination of phenolic compounds, based on high performance liquid chromatography coupled with diode array detector and mass spectrometry using the electrospray ionization interface (HPLC-PDA-ESI-MS) was optimized and validated. Furthermore, the total phenols content was assessed by the Folin-Ciocalteau method, while the antioxidant activity was evaluated by four different assays: ferric reducing antioxidant power (FRAP), oxygen radical absorbance capacity (ORAC), dyphenyl-1-picrylhydrazyl (DPPH) radical scavenging, and Trolox equivalent antioxidant capacity (TEAC).

The above-mentioned methods were applied to analyze the phenolic profile of thirty samples of monovarietal and multivarietal EVOOs samples including PDO oils from different areas in Italy (Sicily, Lazio, Tuscany, and Puglia) and to investigate the difference in total phenolic content and antioxidant activity. To the best of our knowledge, there is no report about the evaluation of antioxidant capacity, employing the four selected different assays, and phenolic compounds profile of EVOOs produced in Italy in different regions.

## 2. Results and Discussion

### 2.1. High-Performance Liquid Chromatography Method Optimization and Validation

Method optimization was carried out analyzing a mixture of representative phenolic compounds that could be present in EVOOs, namely 4-hydroxyphenylacetic acid, p-coumaric acid, vanillic acid, oleuropein, apigenin, pinoresinol, luteolin, and 2-(4-hydroxyphenyl)ethanol. A column containing pentafluorophenyl (PFP) stationary phase (SP) was used for their separation applying a gradient elution mode. The selected stationary phase exhibits moderate hydrophobic properties where a reversed–phase mechanism takes place. In addition, interactions such as hydrogen bonding, dipolar, and steric, have to be considered. Therefore, considering the physical-chemical properties of the studied phenolic compounds, this SP was selected in this study.

Preliminary results utilizing either methanol or acetonitrile alone did not allow to obtain best separation, while their combination, in presence of an acidic modifier (formic acid), enabled the separation of all analytes in reasonable time and with good resolution. Separated peaks were recorded with a PDA detector and the signal extracted at 280 nm because the highest sensitivity.

Method validation was assessed studying some parameters such as repeatability of retention time, limit of detection (LOD), limit of quantitation (LOQ), linearity range, and recovery analyzing the eight standard phenolic compounds.

The intra-day repeatability, calculated after measuring the retention time (*t_R_*) (five runs), was very good with RSDs in the range 0.4–0.8%, while the inter-day RSDs were between 0.3 and 0.8%. Peak area precision, intra-day, and day-to-day resulted in being in the range of 5.9–10.2% and 5.8–10.5%, respectively. RSD% values are acceptable and in accordance to the ones obtained for other published EVOO phenolic compounds analysis methods [32,33]. Linearity was studied analyzing a standard mixture containing 4-hydroxyphenylacetic acid, p-coumaric acid, vanillic acid, oleuropein, apigenin, pinoresinol, luteolin, and 2-(4-hydroxyphenyl)ethanol at concentration range for each analyte of LOQ-140 mg·L^−1^ without an internal standard. Reporting peak areas vs. analytes concentration, a good correlation coefficient was obtained (R^2^ between 0.989 and 0.998). Recovery was verified analyzing sample solutions containing 10 and 50 µg g^−1^ of each standard compound recording good results (73.9–92.1% and 79.1–102.8%, respectively). LOD and LOQ values were below 1 mg·L^−1^ and 2 mg·L^−1^, respectively. Data related to method validation about the studied phenolic compounds are reported in details in Table S1.

In order to tentatively identify the separated compounds in real EVOOs samples, the HPLC instrument was connected to a mass spectrometer and MS spectra acquired.

### 2.2. Quantification of Phenolic Compounds in EVOO Sample and Their Identification

**Figure 1:**
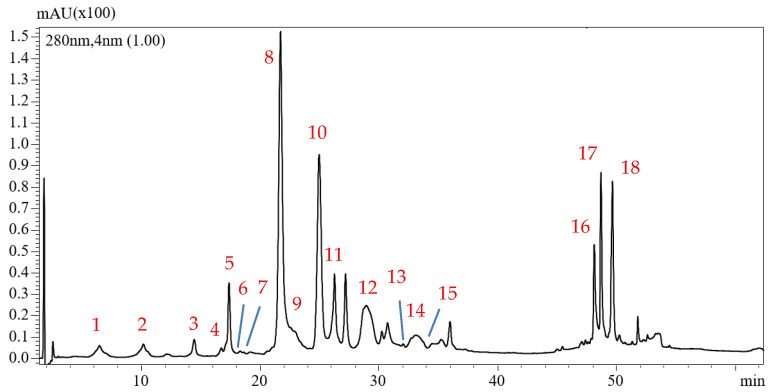
HPLC-PDA chromatogram of phenolic compounds present in one of the analyzed EVOO sample. **1**: Hydroxytyrosol, **2**: Tyrosol, **3**: Vanillic acid, **4**: Ligstroside derivative, **5**: p-cumaric acid, **6**: Hydroxydecarboxymethyl elenolic acid, **7**: Elenolic acid, **8**: Dialdehydic form of oleuropein aglycon, **9**: Oleuropein aglycon, **10**: Oleuropein aglycon. **11**: Lygstroside aglycon, **12**: Oleuropein aglycon, **13**: Luteolin, **14**: lygstroside aglycon, **15**: Apigenin, **16**: Unknown, **17**: Unknown, and **18**: Unknown.

The HPLC analysis of a real EVOO sample revealed the presence of eighteen peaks that could be ascribed to phenolic compounds belonging to different classes. Among them, fifteen were tentatively identified (see Figure 1). The identification was done considering: *t_R_* and UV spectra data, MS spectra, use of standard compounds and data available in literature. As can be observed the oil extract contained phenolic acids, phenolic alcohols, seicoridoids, and flavonoids. Among them, compounds **9**, **10**, and **12** (three isomers of oleuropein aglycon (3,4-DHPEA)) and compounds **11** and **14** (two isomers of ligstroside aglycon), possessing the same mass, were separated because of the chromatographic method.

Afterwards the studied analytical method was applied to the analysis of thirty oil samples of different origin (Sicily, Lazio, Tuscany, and Puglia, Italy) and cultivar. Table S2 reports the geographical origin of the analyzed EVOO sample oils.

A LLE procedure was applied to the analyzed oils in order to obtain sample solutions for the HPLC analysis. After dissolving the samples in hexane, a mixture solution containing MeOH/H_2_O (3:2, *v*/*v*) was added to extract the phenolic compounds. Next hexane was added and this fraction discarded to eliminate the lipid components present in the sample. After evaporation of the aqueous/MeOH, compounds were dissolved in MeOH/H_2_O (1:1, *v*/*v*) and injected for the analysis. The qualitative profile of the phenolic compounds contained in the analyzed oils was quite similar, however, some differences related to peak area were observed.

**Figure 2:**
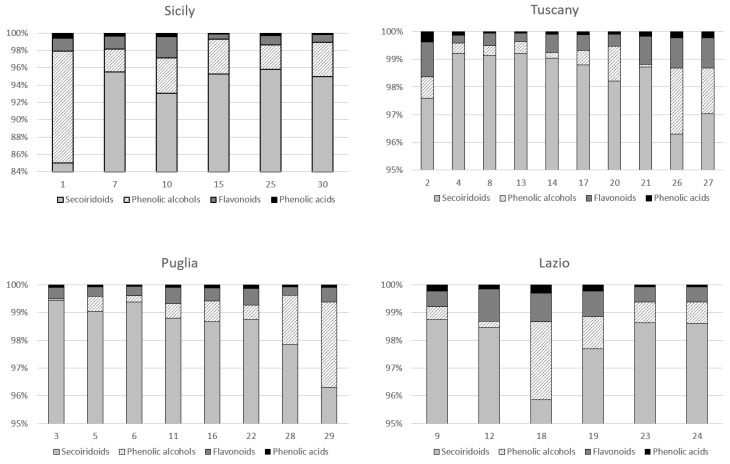
Percentage distribution of phenolic compounds classes (phenolic acids, phenolic alcohols, secoiridoids, and flavonoids) for each analyzed sample in the four different Italian areas (Sicily, Tuscany, Lazio, and Puglia). Sample number refers to Table S2.

Table S3 presents the results of the quantitative analysis of the phenolic compounds present in all analyzed samples, while Figure 2 shows the percentage results of the phenolic compounds according to the different classes present in the studied oil varieties. As can be observed, in all samples of different geographical origin the same compounds have been found. They include the following phenolic classes: secoiridoids (compounds **4**, **6**–**11**, and **14** of the chromatogram), phenolic alcohols (#1 and 2), flavonoids (**13** and **15**) and phenolic acids (**3** and **5**). Three unknown compounds were also found in the extracts. Nine different secoiridoid derivatives were detected and tentatively identified as being the dialdehydic form of oleuropeine aglycon, seven aglycon isomers of oleuropeinand ligstroside, elenolic acid, and a derivative of elenolic acid. During oil extraction β-glucosidase enzymes are responsible for hydrolysis of oleuropein, demethyloleuropein, and ligstroside generating aglycon forms of the secoiridoid glucosides.

In all samples secoiridoids were the main compounds ranging from 85 to more than 99% (*w*/*w*) of the total concentration of detected phenolic compounds according with results of Bakhouche et al. [34]. These results are in agreement with previous studies, which report secoiridoid derivatives as the most abundant VOO phenolic compounds [35,36]. Two flavonoids and two phenolic alcohols were detected, namely luteolin and apigenin, and tyrosol and hydroxytyrosol, respectively. Hydroxytyrosol and tyrosol are simple alcohols conjugated to form oleuropein derivatives, while luteolin and apigenin are the two most representative flavonoids found in VOO. Their percentage ranged from 0.3 to 1.5% (*w*/*w*) and from 0.1 to 12% (*w*/*w*) for flavonoids and phenolic alcohols, respectively. Vanillic and p-coumaric acids, belonging to the class of phenolic acids, were detected. The content was in the range 0.1–0.6% (*w*/*w*) of total concentration of analyzed compounds. These results are in accordance with literature data [4,34].

Table 1 shows the mean, minimum and maximum concentration (expressed as mg kg^−1^ of oil) of phenolic compounds found in EVOO samples produced in the four different Italian areas. The sum of individual concentrations of studied phenolic compounds ranged from 814 to 5920 mg kg^−1^ of oil with a mean value of 3049 mg kg^−1^. Variability was observed both among samples from different Italian areas and samples produced in the same geographical area in accordance with literature data [4]. 

Secoiridoid total concentration, calculated as the sum of concentrations of individual secoiridoids, ranged between 2671 and 5034 mg kg^−1^, 1088 and 5077 mg kg^−1^, 1276 and 3294 mg kg^−1^, 471 and 1577 mg kg^−1^, for Puglia, Tuscany, Lazio, and Sicily, respectively. The ranges and mean values for the different geographical areas show two groups Puglia and Tuscany with higher values and Lazio and Sicily with lower ones. Among secoiridoids, the dialdehydic form of oleuropein aglycon (3,4-DHPEA-EDA), oleuropein, and ligstroside and aglycone isomers showed the highest concentrations ranging from 88 to 1547 mg kg^−1^, from 22 to 1683 mg kg^−1^, from 81 to 900 mg kg^−1^, respectively, for all analyzed samples. Tyrosol and hydroxytyrosol concentrations ranged between 0.03 and 62 mg kg^−1^, and 0.7 and 63 mg kg^−1^, respectively. Luteolin and apigenin concentrations did not show a considerable variability respect to the other classes ranging between 12 and 17 mg kg^−1^, and 1 and 3 mg kg^−1^, respectively. Concentrations of vanillic and p-coumaric acids were below 4 mg kg^−1^ for all analyzed samples with low variability.

It is well known that EVOO phenolic compounds concentration is affected by the olive cultivar, the maturation stage, geographical region, and extraction conditions, as well as the agricultural practices [37]. Moreover, analytical methods, including phenolic compounds extraction, as well as the number of quantified compounds, which include a number of secoridoid derivatives and isomers, deeply influence the obtained results [38]. For these reasons, it is difficult to compare obtained results with that one’s reported in the literature because of no official method, which would be practical and would guarantee the comparison of results exists [39]. Recently, Olmo-Garcia et al. applied different strategies mostly employed for the analisys of EVOO phenolic compounds like LC-MS method, Folin-Ciocalteau assay, the International Olive Council (IOC) method, and hydrolysis plus HPLC-DAD for the analysis of 50 samples. They demonstrated that total phenolic content (summing individual concentration of detected compounds) proved to be higher (1.9–3.0 times when data was expressed in mg/kg) than the values given by the three non-specific methods. They reported that secoiridoids concentration ranged from 127.5 to 2327.7 mg kg^−1^, these results are in agreement with our data also considering that we included in the secoridoids group, as well as elenoic acid and their derivatives, while they considered these last compounds separately [40].

Based on published results on the analysis of some Italian oils the total concentration of phenolic compounds determined by liquid chromatography method values are lower than that ones reported by Olmo-Garcia et al. for example between 260–613 mg kg^−1^ [26], between 41 and 167 mg kg^−1^ [27], between 172 and 326 mg kg^−1^ [41], and between 336 and 978 mg kg^−1^ [30].

These observed difference could be due to all the consideration already described. Moreover higher average values obtained for some samples could also be due to the formation of “artificial peaks” (secoiridoids’ isomeric forms) when using MeOH as extraction solvents and mobile phase, as reported in recently published papers. For this reason the concentration of secoridoids could be underestimated if these peaks are not considered [42,43]. Moreover we included in our quantification three unknown compounds which were not identified but tentatively attributed to the phenolic compounds class based on their UV spectra.

Comparing the different Italian geographical areas the highest total phenolic compounds concentration, calculated as the sum of concentration of analyzed compounds, was found in the Puglia area. An ANOVA test showed a significant statistical difference between Puglia and Sicily total concentration of phenolic compounds determined by HPLC method. The same results were reported in the paper of Antonini et al. [26] showing a higher concentration of phenolic compounds from Puglia respect to Sicily. Although statistically significant differences were detected between the regions of Sicily and Puglia, it was not possible to cluster the samples on a geographic basis, probably because there are many factors influencing the variability of phenolic compounds in EVOO, in particular the type of cultivar, as reported in the literature [44].

### 2.3. Antioxidant Activity of Sample Extracts

The antioxidant ability of the total phenolic compounds content of the thirty samples was evaluated with four well known different chemical assays: TEAC and DPPH radical scavenging tests, FRAP test and ORAC test. Trolox was used as reference compound for all tests and the results were reported as µmol TEg^−1^ of oil. Moreover total phenols concentration (TPC) was measured by the colorimetric Folin-Ciocalteau assay. Table S4 reports the total phenolic compounds concentration measured by HPLC (obtained as the sum of each identified component), TPC determined by Folin-Ciocalteau assay and antioxidant activity values. Table 2 shows ranges values of TPC, TEAC, DPPH, FRAP grouped by geographical areas. Results show that TPC values of all tested samples were between 98 and 573 mg GAE kg^−1^ of oil. TPC values of EVOO samples reported in the literature vary greatly [45]. Ranges reported for Italian EVOOs samples in different papers are in agreement with the obtained results and ranged between 121.3 and 388.0 mg GAE kg^−1^ [29], between 14.8 and 121.2 mg GAE100 g^−1^ [28], between 133.7 and 322.2 μg GAE g^−1^ [46].

The lowest TPC value was for sample #10 from Sicily while the highest one was for sample #4 from Tuscany. Pearson correlation analysis showed a good and significant correlation (R^2^ = 0.893, *p* ≤ 0.001) between total phenolic compounds concentration determined by the two different methods (Table 3).

The DPPH and TEAC assays measure the extracts radical scavenging capacity. TEAC and DPPH values ranged between 2.11 and 8.94 µmol TEg^−1^, and 0.42 and 2.41 µmol TEg^−1^, respectively. FRAP assay measures the capacity of extracts compounds to reduce ferric ions while ORAC assay is frequently used to test the capacity of compounds to protect a fluorescent probe from the oxidative degeneration. FRAP and ORAC values ranged between 0.59 and 3.19 µmol TEg^−1^, and 1.67 and 17.99 µmol TEg^−1^, respectively. Obtained results are in agreement with the ones reported from the analysis of Italian EVOO samples [29,41]. Pearson correlation between total phenolic compounds concentration measured by Folin-Ciocalteau assay and HPLC method and antioxidant activity was evaluated. A very good positive and significant correlation was measured between the antioxidant activity values determined by the four tests and the total concentration of phenolic compounds and among the different antioxidant tests (Table 3). Pearson R^2^ values ranged between 0.607 and 0.893. These results indicated that as the total phenolic compounds concentration increases the antioxidant capacity, measured by tests based on different reaction mechanisms, increases independently from the sample origin.

Variability was observed for total phenolic content and antioxidant activity values both among samples from different areas and among samples of the same area. However, ANOVA test showed a significant difference between antioxidant activities measured by TEAC and ORAC assays between samples from Puglia (highest values) and Sicily (lowest values) as observed also for total phenolic compounds concentration measured by HPLC.

A correlation among the different parameters was investigated in particular, to analyze how the differences recorded by antioxidant activity values determined by different tests can be explained on the basis of the concentrations of the individual phenolic compounds, or, in particular, which classes of molecules were responsible for the antioxidant activity variability. Results showed a very good positive and significant correlation between concentration of oleuropein aglycon and ligstroside aglycon and antioxidant activity determined by the four assays, R^2^ values higher than 0.6 (see Table S5). A good positive and significant correlation even if with lower R^2^ values was determined also between the concentration of dialdeydic form of oleuropein aglycon and oleuropein aglycon isomer and antioxidant activity (see Table S5). These compounds are the most abundant one in almost all the sample and belong to the family of secoiridoids. The high antioxidant activity of these compounds has been demonstrated and described in the literature [5].

## 3. Materials and Methods

### 3.1. Chemicals

Solvents employed for the extraction procedure and for HPLC-MS analyses were ethanol (EtOH) (99.8%) n-Hexane (purity 99.8%), methanol (MeOH) (purity 99.9%), water (HPLC-MS grade), acetonitrile (purity 99.9%), dimethyl sulfoxide (DMSO), and formic acid (purity 95–97%) and were purchased from Sigma-Aldrich (Milan, Italy). The standard compounds namely 4-hydroxyphenylacetic acid (purity 98%), p-coumaric acid (purity 98%), vanillic acid (purity 97%), oleuropein (purity 98%), apigenin (purity 99%), pinoresinol (purity 95%), luteolin (purity 98%), and 2-(4-hydroxyphenyl)ethanol (purity 98%), gallic acid, and Trolox were purchased from Merck KGaA (Darmstadt, Germany).

The reagents as TPTZ (2,4,6-tripyridyl-s-triazine), FeCl_3_·6H_2_O, glacial acetic acid, fluorescein, 2,20-azobis(2-methylpropionamidine) dihydrochloride (AAPH), sodium carbonate, Folin-Ciocalteu reagent, potassium persulfate, ABTS (2,2-azinobis-(3-ethylbenzothiazoline-6-sulfonic) diammonium salt), and DPPH (2,2-dyphenyl-1-picrylhydrazyl) were purchased from Merck KGaA (Darmstadt, Germany).

### 3.2. Samples

Thirty mono- and multivarietal and PDO extra-virgin olive oils samples were collected from Italian producers located in different areas belonging to the harvest year 2017. Samples characteristics (geographical origin and varieties) are reported in Table S2. The cultivars were as follows: Sicily: Nocellara del belice, Tonda iblea, Biancolilla, Cerasuola; Lazio: Frantoio, Leccino, Moraiolo, Rajo, Mariolo; Tuscany: Frantoio, Moraiolo, Maurino, Picholine, Pendolino, Correggiolo, Lecino, Olivastra saggese, Mariolo; Puglia: Coratina, Ogliarola, Cima di Bitonto, Cima di Mola. All extra virgin oil samples were kept at 4 °C, away from light.

### 3.3. Extraction of Phenolic Compounds

Phenolic compounds were extracted by liquid-liquid extraction (LLE) according to the procedure reported by Ricciutelli and co-workers, with some modification [47]. Briefly, one gram of oil was dissolved in 1 mL of hexane in a 15 mL centrifuge tube. Then the polyphenols were extracted using a mixture of MeOH/H_2_O (3:2, *v*/*v*), vortexed and centrifuged for 3 min at 3000 rpm. Extraction was carried out with ultrasound bath (Elmasonic S30H, Elma Schmidbauer GmbH, Singen, Germany) at room temperature, frequency of 37 kHz and heating power of 200 W for 20 min.The procedure was repeated four times. A volume of 2 mL of hexane was added to the extracted solution, vortexed and centrifuged for 5 min 3000 rpm, and then hexane was discarded. Solvent was evaporated in a rotary evaporator (Eyela, Tokyo, Japan). The extract was then dissolved in 500 μL of a mixture of MeOH/H_2_O (1:1, *v*/*v*).

### 3.4. Determination of Total Phenolic Content

Determination of total polyphenol content of the olive oil extracts was determined by the Folin-Ciocalteau method, according to the method described by Singleton and co-workers [48]. To 20 µL of sample extracts (diluted two times) were added 1580 µL of a MeOH/H_2_O (50:50, *v*/*v*) mixture. The blank was prepared in 1600 µL of a MeOH/H_2_O (50:50, *v*/*v*). Then 100 µL of Folin-Ciocalteu reagent was added. The mixture was stored at room temperature in the dark for 8 min. Then 300 µL of sodium carbonate solution (20% *w*/*v*) was added, mixed and incubated for 2 h in a dark environment at room temperature, and finally was centrifuged at 20,817× *g* for 5 min. The absorbance reading was 765 nm in a 96-well cell culture plate (Greiner Bio-one, Kremsmünster, Germany) using a multifunctional microplate reader (Infinite^®^, 200 PRO multimode reader, Tecan, Männedorf, Switzerland). The results were expressed as mg of gallic acid equivalents (GAE)g^−1^ of sample. Total phenolic content was determined from the gallic acid calibration curve (0.05–1.6 mg mL^−1^) (y = 0.8008x + 0.0065. R^2^ = 0.9987).

### 3.5. Antioxidant Activity

Antioxidant activity was evaluated by four different assays namely the ferric reducing antioxidant power (FRAP), according to the method described by Li Fu et al. [49]; oxygen radical absorbance capacity (ORAC), according to the method described by Trombetta et al. [41]; 2,2-dyphenyl-1-picrylhydrazyl (DPPH) radical scavenging, according to the method described by Padmanabhan et al. [50]; and Trolox equivalent antioxidant capacity (TEAC), according to a previously described method with some modification [51].

#### 3.5.1. FRAP Assay

The FRAP (ferric reducing antioxidant power) assay, is a colorimetric method based on the reductionof a ferric tripyridyltriazine complex (Fe^+3^-TPTZ) to its ferrous form (Fe^+2^), at low pH [52]. The FRAP assay was carried out according to the procedure described in literature by Fu et al. [49]. Before the analyses, oil extracts were diluted 20 times. The fresh working FRAP reagent was prepared daily by mixing acetate buffer (300 mM sodium acetate, pH 3.6), 2,4,6-tris(2-pyridyl)-S-triazina solution (10 mM in 40 mM HCl) and FeCl_3_·6H_2_O solution (20 mM) at the ratio 10:1:1 (*v/v/v*), respectively. The reagent FRAP was warmed to 37 °C in a water bath for 10 min. A volume of 10 μL of diluted sample solution was added to 190 μL of FRAP reagent. A blank sample was also prepared as described above, 10 μL of ethanol was added instead of the sample. The absorbance was measured after 6 min at room temperature at wavelength of 593 nm in a 96-well cell culture plate (Greiner Bio-one, Germany) using a multifunctional microplate reader (Infinite^®^, 200 PRO multimode reader, Tecan, Männedorf, Switzerland). Each test sample dilution was performed in triplicate. The antioxidant capacity of sample was determined by a calibration curve, using Trolox as reference compound (25–500 µM). Results were expressed in μmol of Trolox Equivalent (TE) g^−1^ of oil.

#### 3.5.2. ORAC Assay

The ORAC assay was performed as described by Trombetta et al. [41], with the following modifications. Fluorescence was read with an excitation wavelength of 485 nm and an emission wavelength of 520 nm. Before the analyses, oil extracts were diluted 500 times. A fluorescein stock solution (70 nM, final concentration) was prepared in 75 mM phosphate buffer (NaH_2_PO_4_-H_2_O and Na_2_HPO_4_-2H_2_O, pH 7.4) aqueous solution. A volume of 120 μL of Fluorescein and 20 μL of diluted extract solutions were mixed and incubated for 15 min at 37 °C. The blank was properly diluted in 75 mM phosphate buffer (pH 7.4) without sample. The reaction was started with the addition of 60 μL AAPH solution (12 mM, final concentration), prepared daily in 75 mM phosphate buffer (pH 7.4). The volume of the final reaction mixture was 200 μL. The microplate was immediately placed in the reader and the fluorescence decay was measured every minute for 90 cycles in a 96-well cell culture plate (Greiner Bio-one, Germany) using a multifunctional microplate reader (Infinite^®^, 200 PRO multimode reader, Tecan, Männedorf, Switzerland). The microplate was automatically shaken prior each reading.

The area under the curve (AUC) of the blank and samples, due to the decay curves of fluorescein, were calculated by One-way analysis of variance (ANOVA) followed by area under curve using GraphPad Prism version 4 for Windows (GraphPad software, versio 4, San Diego, CA, USA). The net AUC was calculated by subtracting the AUC of the blank to the sample. The activity of the sample was expressed as μmol TE g^−1^ of oil. The antioxidant capacity of sample was determined by a calibration curve, using Trolox as reference compound (0–100 µM) (y = 14697x + 32669; R^2^ = 0.997).

#### 3.5.3. DPPH Radical Scavenging Assay

The DPPH assay was performed according to the method described by Padmanabhan and Jangle [50], with a few modifications. Before the analyses, oil extracts were diluted 20 times. A volume of 20 µL of diluted sample and 180 µL of ethanolic solution of DPPH (0.1 mM) were placed in the well of the microplate. The blank was prepared in ethanol 100%, while the control sample was prepared with 20 µL of ethanol and 180 µL of DPPH (0.1 mM). After 20 min of incubation, in the dark at room temperature, the absorbance was measured at 518 nm in a 96-well cell culture plate (Greiner Bio-one, Germany) using a multifunctional microplate reader (Infinite^®^, 200 PRO multimode reader, Tecan, Männedorf, Switzerland). The antioxidant capacity of sample was determined by a calibration curve, using Trolox as reference compound (20–200 µM) (y = 0.0015x – 0.0246, R^2^ = 0.995).

#### 3.5.4. TEAC Assay

Free radical scavenging activity of oil extract was evaluated by colorimetric assay according to a previously described method with some modification [51]. The ABTS radical (ABTS^+^) was produced by mixing 7 mM ABTS stock solution with 2.5 mM potassium persulfate, in aqueous phosphate buffer (5 mM NaH_2_PO_4_-H_2_O and 5 mM Na_2_HPO_4_-2H_2_O, pH 7.4), for 12 h in the dark at room temperature. The ABTS^•+^ was diluted in phosphate buffer (pH 7.4, 5 mM) to obtain an absorbance of 0.70 ± 0.05 at 734 nm. Before the analyses, extracts were diluted 60 times. A volume of 10 µL of each diluted sample was mixed with 190 µL of ABTS^•+^ working solution in a 96-multiwell plate (Greiner Bio-one, Kremsmünster, Germany). The absorbance was recorded at 734 nm after 20 min using a multifunctional microplate reader (Infinite^®^, 200 PRO multimode reader, Tecan, Männedorf, Switzerland). A calibration curve was prepared with Trolox at the concentration range of 50–700 µM. Results were expressed as µmol TEg^−1^ of oil (y = 0.0009x + 0.0158; R^2^ = 0.998).

### 3.6. HPLC-DAD-ESI-MS Analysis of Phenolic Compounds

The analyses were carried out on a Shimadzu Prominence LC-20A instrument (Shimadzu, Milan, Italy) equipped with two LC-20 AD XR pumps, SIL-10ADvp, CTO-20 AC column oven and DGU-20 A3 degasser coupled to a SPD-M10Avp PDA detector and a mass spectrometer detector (LCMS-2010, Shimadzu, Tokyo, Janpan) equipped with ESI interface. MS data were acquired by Shimadzu LCsolution Ver. 3.7 software (Shimadzu, Tokyo, Janpan). Phenolic compounds separation was carried out using a PFPColumn (150 × 2.1 mm I.D., 2.7 μm d.p.) (Merck KGaA, Darmstadt, Germany). Elution was performed at 40 °C with H_2_O/0.1% HCOOH (solvent A) and methanol/acetonitrile at 1:1 (*v*/*v*)/0.1% HCOOH (solvent B) at a constant flow–rate of 0.2 mL/min. The gradient elution profile was as follows: 0–5 min 5% B, 5–15 min 5–30% B, 15–40 min 30–50% B, 40–50 min 50–100% B. The injection volume was 2 μL. Data were acquired using a PDA in the range 210–400 nm and the chromatograms were extracted at 280 nm. Chromatograms were acquired in the MS instrument using ESI as interface in negative ionization mode using the following parameters: nebulizing gas flow (N_2_): 1.5 mL·min^−1^; Event time: 1 s; mass spectral range: 100–800 *m*/*z*; scan speed: 1000 amu/sec; detector voltage: 1.5 kV; Interface temperature: 250 °C; CDL temperature: 300 °C; heat block: 300 °C; interface voltage: −3.50 kV; Q-array: 0.0 V; Q-array RF: 150.0 V.

### 3.7. HPLC-DAD Quantitative Analysis Method Validation

Standard compounds were chosen, according to the phenolic composition of EVOO oils. A mixture of eight phenolic standard compounds (4-hydroxyphenylacetic acid, p-coumaric acid, vanillic acid, oleuropein, apigenin, pinoresinol, luteolin, and 2-(4-hydroxyphenyl)ethanol) was employed for quantitative analysis and method validation. The analytical method was validated considering linearity, repeatability (intra-day and inter-day precision), limit of detection (LOD), and limit of quantification (LOQ).

Linearity was determined by the calibration curves obtained from the HPLC analysis of the standard solutions. Stock standard solutions of each compound were prepared at a concentration of 10,000 mg·L^−1^ in MeOH for 4-hydroxyphenylacetic acid, p-coumaric acid, vanillic acid, 2-(4 hydroxyphenyl)ethanol) and oleuropein; at a concentration of 10,000 mg·L^−1^ in DMSO for pinoresinol; at a concentration of 2500 mg·L^−1^ in DMSO for luteolin, and at a concentration of 2000 mg·L^−1^ in EtOH for apigenin.

### 3.8. Statistical Analysis

Analyses were performed all in triplicate. Differences were evaluated by one-way analysis of variance (ANOVA) followed by a Bonferroni post-hoc test, if applicable, after verifying the normality of the distribution of the dependent variable. Correlations where evaluated using Pearson correlation analysis, after verifying the normality of the distribution of the variables. Probability values of *p* ≤ 0.05 were considered statistically significant. All statistical analysis where conducted using Stata 14 statistical software (StataCorp LLC, College Station, TX, USA).

## 4. Conclusions

A HPLC-PDA/ESI-MS method has been developed and validated for the analysis of the most representative phenolic compound in EVOO samples. Solvent extraction, using a small amount of oil samples, was employed for the extraction of phenolic compounds. After full validation the method was applied to the analysis of selected phenolic compounds in thirty DPO EVOO samples from four different Italian areas. Extracts’ antioxidant activity was measured using four chemical assays based on different reaction mechanisms and substrates. The approach allowed assessing a qualitative and quantitative analysis of the principal EVOOs phenolic compounds. Quantitative results showed differences with data reported in the literature. This result was discussed considering the challenges in the analysis of these compounds in a well-known food matrix like EVOO. Correlations among total and individual concentration of phenols and samples antioxidant activity was verified. A very good and significant correlation was determined among phenolic compounds concentration and antioxidant activity using assays based on different chemical reaction. Differences in phenols concentration and antioxidant activity were evidenced between samples from two of the four studied geographical areas.

## Figures and Tables

**Table 1:** Mean, minimum, and maximum concentration (expressed as mg kg^−1^ of oil) of phenolic compounds found in EVOO samples produced in the four different Italian areas.

		SICILY mg kg^−^^1^			PUGLIA mg kg^−^^1^			TUSCANY mg kg^−^^1^			LAZIO mg kg^−^^1^		
Compounds	[M − H]^−^ (*m*/*z*)	Range	Average	SD	Range	Average	SD	Range	Average	SD	Range	Average	SD
**Phenolic alcohols ^a^**													
Hydroxytyrosol	153	9.20–62.7	36.68	20.73	1.30–53.41	21.76	20.02	0.72–38.64	13.49	11.44	2.61–24.43	14.50	8.02
Tyrosol	137	0.03–62.30	23.08	22.81	0.32–41.88	12.77	17.35	0.88–14.02	4.96	4.42	0.34–12.83	6.01	5.46
All		20.45–125.00	59.76	70.27	1.90–95.29	34.54	107.51	1.7–47.68	18.45	73.38	3.40–37.26	20.51	57.26
**Phenolic acids**													
Vanillic acid ^b^	167	0.94–2.90	1.68	0.77	1.07–2.50	1.90	0.49	0.71–3.02	1.65	0.80	0.98–3.06	1.87	0.85
p-cumaric acid ^c^	163	0.99–3.10	1.75	0.70	1.26–1.49	1.36	0.12	1.20–3.66	1.70	0.72	1.19–2.36	1.68	0.37
All		2.01–3.93	3.44	41.63	2.33–3.85	3.27	15.78	2.13–6.68	3.35	39.15	2.31–5.42	3.56	32.41
**Secoiridoids ^d^**													
Elenolic acid	241	1.99–30.75	10.59	10.60	2.21–12.13	6.31	3.71	1.63–17.90	7.24	4.99	1.41–9.92	5.46	3.56
Hydroxydecarboxymethyl elenolic acid	199	5.10–41.70	18.00	13.96	2.90–16.51	7.94	4.37	2.29–22.83	9.45	6.01	2.59–27.93	12.94	9.22
Hydroxydecarboxymethyl oleuropein aglycon	335	11.36–67.90	23.08	22.08	3.06–18.24	12.31	5.11	8.59–19.57	12.49	3.65	11.70–24.29	18.38	4.83
dialdehydic form of oleuropein aglycon (3,4-DHPEA-EDA)	319	93.10–992.77	374.83	319.20	278.00–839.10	619.01	171.14	87.7–1235.24	689.43	385.35	332.08–1546.61	840.17	418.75
Oleuropein aglycon (3,4-DHPEA)	377	20.41–63.81	36.99	17.02	104.95–387.61	187.20	110.34	30.72–923.83	294.75	330.87	26.84–135.81	59.29	41.85
Oleuropein aglycon	377	164.67–655.31	347.58	180.26	333.70–996.01	631.16	216.29	156.9–660.10	479.55	151.13	390.09–912.35	565.01	209.01
Ligstroside aglycone (p-HPEA-EA)	361	80.83–180.11	126.06	43.32	205.90–786.98	434.66	235.19	124.36–899.48	326.90	245.74	152.81–218.13	188.14	24.97
Oleuropein aglycon	377	21.89–375.80	147.79	122.33	747.46–1682.89	1163.66	385.34	233.93–1468.91	746.51	489.56	160.89–623.65	354.40	196.96
Ligstroside aglycone (p-HPEA-EA) isomer	361	23.03–216.15	85.38	68.49	324.35–990.64	591.86	23.39	84.20–569.69	247.05	148.29	106.82–412.32	191.93	120.60
All		470.89–2162.66	1170.34	52.73	2670.68–5033.75	3654.14	22.90	1087.60–5300.79	2813.40	55.37	1276.01–3296.84	2235.75	37.44
**Flavonoids**													
Apigenin ^e^	269	1.05–1.04	1.22	0.15	1.21–2.19	1.66	0.29	1.20–2.57	1.78	0.45	1.42–2.91	1.98	0.56
Luteolin ^f^	285	11.58–13.07	12.34	0.69	12.98–16.82	14.12	1.28	12.57–15.47	13.82	0.93	12.17–15.45	14.25	1.59
All		12.64–14.30	13.57	6.00	140.19–18.65	15.79	8.76	13.91–17.35	15.60	8.13	13.9–18.36	16.24	11.55
**Unknown ^d^**													
Unknown 1	411	21.33–188.10	88.28	61.32	25.32–141.19	106.50	34.98	19.22–164.89	93.03	53.36	22.42–144.63	99.92	52.95
Unknown 2	292	25.24–182.70	101.76	74.27	25.38–320.7	125.98	117.61	16.53–271.78	118.08	87.14	21.51–368.49	182.33	147.61
Unknown 3	324	128.10–270.96	205.26	49.08	67.42–491.75	259.56	138.14	73.93–286.62	181.48	62.06	120.39–274.37	165.33	59.09
All		307.99–474.99	395.30	17.11	379.83–672.58	492.05	19.58	159.60–577.81	392.60	27.46	261.14–650.01	447.58	38.16
Bioactive molecules		813.98–2625.01	1642.41		3260.88–5613.61	4199.79		1552.30–5920.33	3243.40		1592.28–3824.12	2723.63	

^a.^ For quantitative determination of all the identified phenolic compounds, the calibration curve of tyrosol was calculated. ^b.^ For quantitative determination of all the identified phenolic compounds, the calibration curve of vanillic acid was calculated. ^c.^ For quantitative determination of all the identified phenolic compounds, the calibration curve of p-cumaric acid was calculated. ^d.^ For quantitative determination of all the identified phenolic compounds, the calibration curve of oleuropein was calculated. ^e.^ For quantitative determination of all the identified phenolic compounds, the calibration curve of apigenin was calculated. ^f.^ For quantitative determination of all the identified phenolic compounds, the calibration curve of luteolin was calculated.

**Table 2:** Mean, minimum, and maximum TPC, TEAC, DPPH, and FRAP values of analyzed EVOO samples grouped by geographical areas.

Geographical Area	TPC (mg GAE kg ^−1^)			TEAC (µmol TE g ^−1^)			DPPH (µmol TE g ^−1^)			ORAC (µmol TE g ^−1^)			FRAP (µmol TE g ^−1^)		
	Range	Avarage	SD	Range	Avarage	SD	Range	Avarage	SD	Range	Avarage	SD	Range	Avarage	SD
Sicily	97.63–236.41	159.06	45.21	2.11–4.59	3.58	0.88	0.53–1.03	0.68	0.20	1.67–10,65	4.50	3.19	0.59–1.18	0.94	0.20
Puglia	268.63–509.00	335.16	112.86	4.83–8.02	6.49	1.31	0.89–1.55	1.13	0.22	6.70–14.69	10.93	2.70	0.80–2.77	1.75	0.67
Tuscany	171.16–573.20	348.25	148.54	3.27–8.94	5.09	2.00	0.57–2.41	1.16	0.65	5.61–17.99	9.46	4.12	0.83–3.19	1.63	0.84
Lazio	161.82–298.23	238.51	70.12	2.91–6.21	4.26	1.27	0.62–1.01	0.85	0.17	2.99–8.37	6.01	2.24	0.96–2.07	1.45	0.42

**Table 3:** Pearson correlation coefficient among total phenolic compounds concentration determined by HPLC method, TPC, TEAC, DPPH, ORAC, and FRAP values.

	Total Phenolic Compounds Concentration HPLC	TPC	TEAC	DPPH	ORAC	FRAP
Total phenolic compounds concentration HPLC	1					
TPC	0.893 ***	1				
TEAC	0.812 ***	0.817 ***	1			
DPPH	0.8659 ***	0.790 ***	0.658 ***	1		
ORAC	0.798 ***	0.706 ***	0.789 ***	0.607 ***	1	
FRAP	0.873 ***	0.836 ***	0.801 ***	0.863 ***	0.649 ***	1

*** *p* < 0.001.

## Supplementary Materials

The following are available online. Table S1. Analytical parameters of proposed method for the analysis of phenolic compounds in EVOOs; Table S2. Geographical origin and olive varieties of the thirty olive oils samples; Table S3. Concentrations of phenolic compounds in thirty samples of EVOOs; Table S4. TPC, TEAC, DPPH, FRAP, and ORAC values of thirty analyzed samples grouped by areas; Table S5. Pearson correlation analysis coefficients of individual phenolic compounds concentration and antioxidant activity values.
