# Supplementary material for: Extraction, Analysis, and Antioxidant Activity Evaluation of Phenolic Compounds in Different Italian Extra-Virgin Olive Oils
Source: Molecules. 2018 Dec 8;23(12):3249. doi: 10.3390/molecules23123249 (PMC6321326; doi:10.3390/molecules23123249)
Supplement: Supplementary file 1 [file molecules-23-03249-s001.pdf]

Table S1: Analytical parameters of proposed method for the analysis of phenolic compounds in extra virgin olive oils.

| **Compounds** | **Linearity** | **Run-to-Run Precision (RSD% n=5)** | | **Day-to-Day Precision (RSD% n=10)** | | **LOD Mg $L^{-1}$** | **LOQ Mg $L^{-1}$** | **Recovery %** | | | |
|---|---|---|---|---|---|---|---|---|---|---|---|
| | $R^2$ | tr | Peak Area | tr | Peak Area | | | **10 ($\mu g\ g^{1}$)** | **RSD %** | **50 ($\mu g\ g^{-1)}$** | **RSD %** |
| Tyrosol | 0.996 | 0.5 | 6.2 | 0.8 | 5.8 | 0.1 | 0.8 | 91.4 | 2.1 | 92.1 | 1.6 |
| 4-hydroxyphenylacetic acid | 0.998 | 0.4 | 5.9 | 0.4 | 6.4 | 0.1 | 0.5 | 86.3 | 2.0 | 93.1 | 3.8 |
| Vanillic acid | 0.995 | 0.4 | 6.6 | 0.4 | 7.9 | 0.001 | 0.05 | 87.9 | 1.3 | 91.2 | 2.1 |
| p-coumaric acid | 0.995 | 0.5 | 6.4 | 0.3 | 7.2 | 0.0001 | 0.001 | 86.4 | 1.4 | 90.4 | 0.8 |
| Oleuropein | 0.995 | 0.6 | 6.5 | 0.4 | 10.5 | 1 | 2 | 73.9 | 15.8 | 79.1 | 3.6 |
| Pinoresinol | 0.993 | 0.7 | 7.4 | 0.5 | 8.2 | 0.05 | 0.1 | 88.1 | 0.3 | 91.4 | 1.1 |
| Luteolin | 0.989 | 0.6 | 7.3 | 0.5 | 8.9 | 0.1 | 0.5 | 85.5 | 1.8 | 95.8 | 6.6 |
| Apigenin | 0.997 | 0.7 | 10.4 | 0.6 | 9.6 | 1 | 2 | 92.1 | 2.6 | 102.8 | 2.0 |

Table S2: geographical origin and olive varieties of the thirty EVOO samples.

| **Sample** | **Geographical origin** | **Variety** |
|---|---|---|
| 1 | Sicily | Monocultivar |
| 2 | Tuscany | Monocultivar |
| 3 | Puglia | Monocultivar |
| 4 | Tuscany | Blend |
| 5 | Puglia | Blend |
| 6 | Puglia | Monocultivar |
| 7 | Sicily | Monocultivar |
| 8 | Tuscany | Blend |
| 9 | Lazio | Blend |
| 10 | Sicily | Monocultivar |
| 11 | Puglia | Monocultivar |
| 12 | Lazio | Blend |
| 13 | Tuscany | Blend |
| 14 | Tuscany | Monocultivar |
| 15 | Sicily | Blend |
| 16 | Puglia | Monocultivar |
| 17 | Tuscany | Monocultivar |
| 18 | Lazio | Monocultivar |
| 19 | Lazio | Monocultivar |
| 20 | Tuscany | Blend |
| 21 | Tuscany | Blend |
| 22 | Puglia | Monocultivar |
| 23 | Lazio | Blend |
| 24 | Lazio | Blend |
| 25 | Sicily | Monocultivar |
| 26 | Tuscany | Blend |
| 27 | Tuscany | Blend |
| 28 | Puglia | Blend |
| 29 | Puglia | Blend |
| 30 | Sicily | Blend |

Table S3: Concentration of phenolic compounds in the analyzed thirty EVOO samples.

| | Hydroxytyrosol | Tyrosol | Vanillic acid | Lingstroside derivative | p-cumaric acid | Hydroxydecarboxymethyl elenolic acid | Elenolic acid | dialdehydic form of oleuropein aglycon | Oleuropein aglycon | Oleuropein aglycon | Ligstroside aglycon | Oleuropein aglycon | Luteolin | Ligstroside aglycon | Apigenin | Unknown | Unknown | Unknown |
|---|---|---|---|---|---|---|---|---|---|---|---|---|---|---|---|---|---|---|
| Sample Number | mg $Kg^{-1}$ of oil (RSD%) | | | | | | | | | | | | | | | | | |
| 1 | 62.7 (5.4) | 62.3 (1.8) | 2.9 (6.7) | 67.9 (2.8) | 3.1 (5.5) | 7.3 (0.5) | 6.1 (8.9) | 218.1 (6.2) | 35.2 (3.0) | 190.5 (1.8) | 91.5 (1.7) | 108.7 (4.6) | 12.9 (2.4) | 98.6 (1.9) | 1.4 (2.7) | 188.1 (6.6) | 149.9 (1.4) | 128.1 (3.6) |
| 2 | 2.5 (17.0) | 6.0 (4.7) | 2.9 (1.4) | 14.2 (0.8) | 1.2 (0.9) | 5.7 (17.9) | 4.2 (6.8) | 87.7 (7.6) | 46.6 (3.4) | 156.9 (7.1) | 269.6 (6.7) | 328.8 (0.4) | 13.0 (1.5) | 173.9 (6.7) | 1.2 (0.5) | 103.1 (3.6) | 210.5 (3.1) | 124.3 (4.9) |
| 3 | 1.3 (4.0) | 0.6 (34.6) | 2.5 (3.9) | 15.8 (4.8) | 1.3 (3.8) | 2.9 (1.8) | 4.5 (3.9) | 278.0 (2.1) | 199.2 (1.4) | 333.7 (12.4) | 758.9 (5.6) | 1440.0 (9.9) | 13.5 (0.4) | 712.8 (0.2) | 1.8 (3.0) | 108.2 (0.7) | 320.7 (1.1) | 121.6 (2.8) |
| 4 | 14.07 (7.77) | 5.27 (3.80) | 3.02 (4.56) | 19.57 (7.00) | 3.66 (4.03) | 9.27 (8.19) | 10.47 (2.43) | 891.69 (0.29) | 923.83 (4.19) | 542.57 (0.57) | 899.48 (1.48) | 1434.22 (0.17) | 13.82 (4.94) | 569.69 (1.31) | 1.89 (4.25) | 151.26 (3.01) | 271.78 (4.32) | 154.77 (5.04) |
| 5 | 17.65 (0.81) | 5.26 (1.05) | 1.89 (0.05) | 7.35 (0.08) | 1.18 (0.02) | 7.49 (0.63) | 6.47 (0.74) | 541.76 (0.74) | 387.61 (0.91) | 571.91 (2.53) | 786.98 (1.02) | 1340.02 (0.70) | 13.43 (0.74) | 627.84 (2.04) | 1.69 (0.35) | 114.48 (0.30) | 25.38 (0.34) | 327.47 (1.66) |
| 6 | 9.68 (0.30) | 0.32 (2.63) | 1.07 (0.63) | 3.06 (3.17) | 1.26 (0.19) | 5.84 (3.32) | 2.21 (6.61) | 688.74 (1.32) | 327.57 (4.45) | 512.67 (1.98) | 537.24 (0.76) | 1572.99 (1.79) | 13.32 (0.46) | 707.07 (2.52) | 1.38 (1.58) | 101.33 (1.31) | 31.80 (3.74) | 303.90 (4.99) |
| 7 | 21.64 (0.61) | 0.03 (3.55) | 1.20 (0.78) | 18.17 (0.85) | 1.61 (0.16) | 12.80 (2.04) | 3.36 (8.29) | 228.64 (1.07) | 26.16 (0.23) | 281.74 (3.01) | 80.83 (0.86) | 82.74 (0.15) | 11.58 (0.02) | 56.43 (1.16) | 1.06 (0.07) | 80.96 (1.09) | 33.53 (2.86) | 237.68 (0.31) |
| 8 | 11.33 (1.48) | 0.88 (16.18) | 0.71 (2.58) | 8.77 (1.88) | 1.51 (1.20) | 7.17 (0.74) | 1.63 (3.54) | 1235.24 (3.62) | 361.37 (3.14) | 625.75 (0.93) | 263.49 (0.21) | 686.90 (3.93) | 13.40 (0.36) | 212.97 (0.66) | 1.61 (0.01) | 69.14 (3.31) | 16.53 (4.58) | 73.93 (5.61) |
| 9 | 11.30 (1.43) | 0.34 (1.97) | 3.06 (2.31) | 24.29 (0.64) | 2.36 (0.18) | 13.46 (2.22) | 9.54 (0.99) | 997.83 (2.75) | 41.60 (0.46) | 511.34 (1.02) | 165.37 (4.86) | 500.62 (1.67) | 12.22 (0.22) | 159.45 (1.16) | 1.82 (1.02) | 144.63 (1.74) | 351.99 (1.93) | 142.68 (1.70) |
| 10 | 9.20 (3.95) | 11.25 (0.21) | 1.02 (0.97) | 11.36 (3.77) | 0.99 (0.43) | 5.10 (0.33) | 30.75 (0.44) | 93.10 (1.25) | 20.41 (0.80) | 164.67 (0.93) | 100.58 (0.53) | 21.89 (0.50) | 11.59 (0.06) | 23.03 (0.12) | 1.05 (0.55) | 102.16 (0.66) | 25.24 (3.38) | 180.59 (6.62) |
| 11 | 10.10 (5.28) | 7.39 (4.49) | 1.83 (3.88) | 14.58 (3.91) | 1.49 (0.38) | 12.03 (1.14) | 12.13 (1.48) | 584.00 (4.19) | 109.50 (0.63) | 674.05 (0.14) | 234.99 (0.31) | 922.43 (0.02) | 16.82 (0.17) | 652.79 (1.78) | 1.83 (0.66) | 120.33 (0.32) | 73.11 (1.58) | 186.39 (0.51) |
| 12 | 2.61 (5.74) | 0.79 (2.73) | 1.12 (0.36) | 16.84 (4.15) | 1.19 (0.01) | 16.95 (2.36) | 5.46 (12.28) | 536.58 (4.05) | 45.83 (1.14) | 390.09 (1.09) | 218.13 (0.21) | 160.89 (0.21) | 15.45 (0.09) | 156.73 (0.58) | 2.91 (3.85) | 143.56 (0.04) | 368.49 (1.16) | 137.96 (0.43) |
| 13 | 18.94 (0.18) | 2.57 (3.01) | 1.03 (0.08) | 12.97 (0.31) | 1.44 (0.42) | 12.75 (1.72) | 17.90 (1.70) | 1108.04 (4.46) | 820.72 (1.25) | 660.10 (0.65) | 615.10 (2.69) | 1468.91 (0.21) | 13.72 (0.04) | 359.96 (0.56) | 2.43 (0.86) | 106.92 (0.65) | 32.06 (1.78) | 173.67 (0.81) |
| 14 | 2.74 (3.75) | 1.64 (9.14) | 0.93 (0.84) | 11.02 (1.20) | 1.20 (0.13) | 2.29 (3.37) | 3.21 (1.99) | 386.36 (4.12) | 77.25 (0.10) | 589.64 (0.54) | 232.95 (0.24) | 626.50 (0.45) | 13.32 (0.03) | 294.92 (0.88) | 1.51 (0.03) | 144.86 (0.43) | 30.52 (0.42) | 286.62 (0.28) |
| 15 | 57.35 (0.81) | 33.14 (0.58) | 0.94 (0.01) | 12.94 (0.28) | 1.50 (0.24) | 41.70 (0.60) | 1.99 (0.60) | 992.77 (1.13) | 51.07 (0.77) | 655.31 (0.62) | 180.11 (0.23) | 173.64 (0.90) | 13.07 (0.07) | 53.13 (0.87) | 1.20 (0.12) | 109.38 (0.01) | 46.03 (1.67) | 199.74 (0.40) |
| | 14.98 | 7.45 | 2.03 | 11.09 | 1.33 | 8.01 | 4.69 | 565.43 | 124.52 | 710.81 | 339.30 | 828.29 | 12.98 | 347.45 | 1.21 | 125.91 | 54.92 | 491.75 |

| | | | | | | | | | | | | | | | | | | |
|---|---|---|---|---|---|---|---|---|---|---|---|---|---|---|---|---|---|---|
| 16 | (0.61) | (4.66) | (0.04) | (0.12) | (0.23) | (1.68) | (0.31) | (0.26) | (0.44) | (0.90) | (0.60) | (1.50) | (0.06) | (0.60) | (2.04) | (0.95) | (1.12) | (0.71) |
| 17 | 15.32 | 0.96 | 2.08 | 17.14 | 1.36 | 5.02 | 9.22 | 952.84 | 319.99 | 449.88 | 202.80 | 890.28 | 14.75 | 213.35 | 2.57 | 109.50 | 149.49 | 134.11 |
| | (0.43) | (0.64) | (1.04) | (0.06) | (0.05) | (0.33) | (0.36) | (0.16) | (0.45) | (0.27) | (0.45) | (0.91) | (0.01) | (0.97) | (0.25) | (0.86) | (0.65) | (0.33) |
| 18 | 24.43 | 12.83 | 2.37 | 23.32 | 1.60 | 4.13 | 2.76 | 332.08 | 26.96 | 422.33 | 186.64 | 198.18 | 12.17 | 79.61 | 1.73 | 119.24 | 21.51 | 120.39 |
| | (1.58) | (0.02) | (1.18) | (0.09) | (0.05) | (0.19) | (1.32) | (2.00) | (4.08) | (0.45) | (1.23) | (0.36) | (0.08) | (0.83) | (0.36) | (0.54) | (0.05) | (0.41) |
| 19 | 14.24 | 7.14 | 2.40 | 18.93 | 1.66 | 12.60 | 3.67 | 838.27 | 26.84 | 429.26 | 152.81 | 187.21 | 15.11 | 106.82 | 1.60 | 125.34 | 50.46 | 191.39 |
| | (0.57) | (5.02) | (1.75) | (4.50) | (0.25) | (0.90) | (8.33) | (0.99) | (8.21) | (0.17) | (0.24) | (0.17) | (0.22) | (1.60) | (0.05) | (0.69) | (0.29) | (0.17) |
| 20 | 38.64 | 9.04 | 1.79 | 11.71 | 1.62 | 22.83 | 11.57 | 922.84 | 285.84 | 539.36 | 352.06 | 1241.38 | 14.92 | 337.42 | 1.93 | 164.89 | 33.29 | 206.22 |
| | (0.06) | (0.03) | (7.19) | (0.15) | (0.01) | (0.54) | (0.67) | (0.39) | (0.42) | (0.39) | (2.06) | (0.06) | (0.46) | (2.48) | (1.45) | (0.43) | (1.92) | (0.45) |
| 21 | 0.72 | 0.98 | 1.15 | 12.12 | 1.95 | 13.03 | 4.39 | 621.61 | 40.98 | 486.31 | 150.09 | 297.76 | 15.47 | 84.20 | 1.88 | 19.22 | 136.92 | 200.44 |
| | (4.13) | (2.77) | (0.74) | (0.22) | (1.00) | (1.71) | (1.97) | (0.99) | (0.62) | (0.53) | (0.68) | (2.90) | (0.25) | (0.23) | (0.50) | (0.73) | (0.30) | (0.72) |
| 22 | 13.91 | 0.32 | 2.11 | 16.61 | 1.49 | 16.51 | 11.15 | 757.72 | 118.40 | 424.97 | 205.90 | 747.46 | 14.54 | 371.96 | 1.63 | 141.90 | 54.41 | 359.89 |
| | (3.05) | (13.75) | (3.34) | (0.10) | (0.09) | (1.26) | (0.33) | (0.20) | (0.81) | (0.18) | (0.52) | (0.74) | (0.37) | (0.09) | (2.15) | (0.77) | (0.51) | (0.91) |
| 23 | 11.88 | 11.82 | 0.98 | 11.70 | 1.72 | 2.59 | 1.41 | 789.67 | 135.21 | 912.35 | 205.33 | 623.65 | 15.21 | 412.32 | 1.42 | 22.42 | 139.32 | 125.20 |
| | (2.29) | (0.86) | (0.75) | (0.41) | (0.19) | (0.02) | (1.23) | (1.52) | (0.48) | (0.77) | (0.35) | (1.34) | (0.02) | (3.09) | (0.06) | (0.77) | (0.33) | (0.63) |
| 24 | 22.54 | 3.15 | 1.30 | 15.21 | 1.58 | 27.93 | 9.92 | 1546.61 | 79.34 | 724.72 | 200.55 | 455.88 | 15.35 | 236.68 | 2.44 | 44.31 | 162.24 | 274.37 |
| | (1.49) | (7.43) | (0.21) | (0.58) | (0.06) | (4.11) | (0.11) | (0.45) | (0.31) | (1.65) | (0.31) | (0.08) | (0.16) | (0.07) | (0.13) | (0.31) | (0.22) | (2.35) |
| 25 | 29.32 | 6.16 | 2.24 | 15.24 | 1.69 | 27.33 | 12.81 | 387.90 | 25.31 | 412.31 | 126.57 | 123.98 | 12.04 | 64.99 | 1.24 | 21.33 | 182.70 | 270.96 |
| | (0.29) | (3.04) | (0.12) | (0.91) | (0.17) | (2.02) | (0.64) | (1.23) | (0.03) | (0.69) | (0.24) | (0.05) | (0.05) | (0.13) | (0.11) | (0.20) | (0.01) | (0.01) |
| 26 | 22.45 | 8.25 | 1.37 | 8.85 | 1.61 | 12.08 | 5.97 | 370.63 | 40.27 | 327.88 | 124.36 | 233.93 | 12.57 | 112.00 | 1.34 | 25.93 | 138.69 | 228.63 |
| | (1.02) | (0.25) | (0.66) | (0.39) | (0.44) | (0.65) | (1.48) | (0.03) | (0.20) | (2.69) | (0.06) | (0.10) | (0.08) | (0.22) | (0.05) | (0.04) | (0.61) | (0.15) |
| 27 | 8.20 | 14.02 | 1.56 | 8.59 | 1.45 | 4.42 | 3.88 | 317.38 | 30.72 | 417.16 | 159.11 | 256.43 | 13.24 | 112.13 | 1.45 | 35.56 | 161.02 | 232.11 |
| | (0.80) | (3.12) | (0.92) | (1.13) | (0.40) | (0.76) | (0.39) | (0.04) | (0.30) | (0.33) | (0.16) | (0.12) | (0.01) | (0.05) | (0.12) | (2.69) | (0.13) | (0.39) |
| 28 | 53.05 | 39.00 | 2.44 | 18.24 | 1.41 | 5.61 | 7.02 | 839.10 | 104.95 | 996.01 | 389.29 | 1682.89 | 13.45 | 990.64 | 2.19 | 114.56 | 286.34 | 67.42 |
| | (0.16) | (0.58) | (0.33) | (0.19) | (0.38) | (0.91) | (0.42) | (0.78) | (0.72) | (0.76) | (0.20) | (0.41) | (0.07) | (0.78) | (0.60) | (0.39) | (1.11) | (1.32) |
| 29 | 53.41 | 41.88 | 1.35 | 11.78 | 1.47 | 5.13 | 2.33 | 697.39 | 125.91 | 825.19 | 224.69 | 775.19 | 14.99 | 324.35 | 1.59 | 25.32 | 161.19 | 218.11 |
| | (1.20) | (0.13) | (0.39) | (0.22) | (0.17) | (1.19) | (1.11) | (1.16) | (0.86) | (1.11) | (0.02) | (0.35) | (0.72) | (1.57) | (0.21) | (0.26) | (0.25) | (0.45) |
| 30 | 39.89 | 25.60 | 1.78 | 12.90 | 1.65 | 13.77 | 8.56 | 328.50 | 63.81 | 380.98 | 176.81 | 375.80 | 12.87 | 216.15 | 1.39 | 27.75 | 173.17 | 214.53 |
| | (0.40) | (1.92) | (0.16) | (0.01) | (0.09) | (0.42) | (0.75) | (0.29) | (0.03) | (1.69) | (0.21) | (0.19) | (0.06) | (0.10) | (0.30) | (0.07) | (0.03) | (0.10) |

Table S4: TPC, TEAC, DPPH, FRAP values of analyzed EVOO samples grouped by geographical areas. Values are expressed as mean ± SD. Sample number refers to table S2.

| | Sample Number | TPC (mg GAE kg-1 ) | TEAC (µmol TE g-1) | DPPH (µmol TE g-1) | ORAC (µmol TE g-1) | FRAP (µmol TE g-1) | HPLC (mg Kg-1 ) |
|---|---|---|---|---|---|---|---|
| SICILY | **1** | 159.70 ± 16.11 | 3.13 ± 0.56 | 0.59 ± 0.08 | 3.03 ± 0.43 | 1.00 ± 0.16 | 1435.20 ± 2.00 |
| | **7** | 138.67 ± 9.89 | 3.45 ± 0.38 | 0.67 ± 0.10 | 3.28 ± 0.59 | 0.90 ± 0.13 | 4821.47 ± 1.23 |
| | **10** | 97.63 ± 7.10 | 2.11 ± 0.27 | 0.46 ± 0.05 | 1.67 ± 0.19 | 0.59 ± 0.03 | 3093.91 ± 1.53 |
| | **15** | 236.41± 18.10 | 3.86 ± 0.32 | 1.03 ± 0.08 | 10.65 ± 0.58 | 1.03 ± 0.15 | 2707.48 ± 0.65 |
| | **25** | 165.46 ± 24.97 | 4.59 ± 0.74 | 0.76 ± 0.10 | 5.00 ± 0.74 | 0.93 ± 0.09 | 3424.21 ± 0.06 |
| | **30** | 156.50 ± 16.84 | 3.32 ± 0.23 | 0.53 ± 0.06 | 3.34 ± 0.35 | 1.18 ± 0.07 | 2075.92 ± 0.31 |
| PUGLIA | **3** | 419.53 ± 14.44 | 7.18 ± 0.81 | 1.32 ± 0.12 | 9.35 ± 1.18 | 1.82 ± 0.30 | 1552.15 ± 1.67 |
| | **5** | 509.00 ± 37.87 | 8.02 ± 0.61 | 1.55 ± 0.11 | 11.76 ± 0.91 | 2.77 ± 0.09 | 5920.32 ± 0.71 |
| | **6** | 409.75 ± 16.29 | 7.86 ± 1.26 | 1.23 ± 0.07 | 14.69 ± 1.44 | 2.04 ± 0.06 | 4785.86 ± 0.44 |
| | **11** | 334.90 ± 19.01 | 5.82 ± 040 | 0.99 ± 0.06 | 6.70 ± 0.83 | 1.27 ± 0.18 | 813.96 ± 1.40 |
| | **16** | 301.77 ± 14.20 | 5.38 ± 0.30 | 0.96 ± 0.08 | 11.01 ± 0.95 | 0.80 ± 0.12 | 2625.00 ± 0.63 |
| | **22** | 268.63 ± 19.59 | 4.83 ± 0.65 | 0.89 ± 0.10 | 8.30 ± 0.66 | 1.41 ± 0.04 | 2089.20 ± 0.73 |
| | **28** | 136.21 ± 13.47 | 2.50 ± 0.19 | 0.42 ± 0.03 | 6.05 ± 0.54 | 0.94 ± 0.05 | 5613.64 ± 0.28 |
| | **29** | 301.46± 26.97 | 5.24 ± 0.54 | 1.00 ± 0.15 | 11.92 ± 0.60 | 1.35 ± 0.10 | 3511.29 ± 0.15 |
| TUSCANY | **2** | 213.14 ± 15.15 | 4.28 ± 0.23 | 0.75 ± 0.07 | 7.58 ± 0.86 | 0.93 ± 0.08 | 2929.45 ± 4.05 |
| | **4** | 573.20 ± 33.66 | 8.94 ± 1.22 | 2.41 ± 0.15 | 15.17 ± 1.66 | 3.19 ± 0.17 | 4317.37 ± 5.08 |
| | **8** | 488.59 ± 33.67 | 7.48 ± 0.14 | 1.71 ± 0.04 | 8.61 ± 0.53 | 2.43 ± 0.30 | 1180.14 ± 0.57 |
| | **13** | 513.03 ± 40.79 | 6.69 ± 0.82 | 1.78 ± 0.11 | 17.99 ± 1.01 | 2.42 ± 0.14 | 2221.55 ± 0.66 |
| | **14** | 246.80 ± 19.05 | 4.34 ± 0.61 | 0.83 ± 0.07 | 9.46 ± 0.75 | 0.90 ± 0.17 | 5429.22 ± 1.49 |
| | **17** | 338.95 ± 31.47 | 4.40 ± 0.54 | 1.30 ± 0.08 | 10.65 ± 0.58 | 1.57 ± 0.13 | 3652.16 ± 0.67 |
| | **20** | 374.12 ± 36.61 | 5.00 ± 0.45 | 1.25 ± 0.11 | 7.29 ± 0.63 | 2.06 ± 0.12 | 2185.76 ± 0.50 |
| | **21** | 171.16 ± 6.24 | 3.27 ± 0.38 | 0.60 ± 0.04 | 5.61 ± 0.91 | 1.00 ± 0.10 | 4197.36 ± 0.32 |
| | **26** | 407.49 ± 34.86 | 7.61 ± 0.48 | 1.05 ± 0.03 | 13.72 ± 1.51 | 2.51 ± 0.26 | 1676.81 ± 0.54 |
| | **27** | 156.00 ± 10.82 | 3.99 ± 0.39 | 0.57 ± 0.05 | 6.17 ± 1.64 | 0.83 ± 0.07 | 1778.42 ± 0.10 |
| LAZIO | **9** | 287.47 ± 9.35 | 4.79 ± 0.31 | 1.07 ± 0.04 | 7.21 ± 0.30 | 1.71 ± 0.10 | 3592.33 ± 2.11 |
| | **12** | 164.64 ± 11.11 | 3.47 ± 0.34 | 0.75 ± 0.04 | 4.68 ± 0.87 | 0.96 ± 0.05 | 3635.78 ± 1.02 |
| | **18** | 161.82 ± 15.60 | 2.91 ± 0.32 | 0.62 ± 0.05 | 2.99 ± 0.43 | 1.04 ± 0.11 | 3490.66 ± 0.30 |
| | **19** | 202.88 ± 20.62 | 3.25 ± 0.28 | 0.82 ± 0.07 | 4.55 ± 0.74 | 1.33 ± 0.10 | 1592.28 ± 0.63 |
| | **23** | 316.04 ± 28.41 | 6.21 ± 0.38 | 0.82 ± 0.05 | 8.26 ± 1.52 | 1.57 ± 0.19 | 2425.75 ± 0.07 |
| | **24** | 298.23 ± 28.96 | 4.93 ± 0.49 | 1.01 ± 0.12 | 8.37 ± 1.37 | 2.07 ± 0.20 | 3260.89 ± 0.30 |

**Table S5:** Pearson correlation coefficients of individual phenolic compounds concentration and antioxidant activity values.

| Compounds | TEAC | DPPH | FRAP | ORAC |
|---|---|---|---|---|
| Hydroxytyrosol | -0.217 | -0.169 | -0.093 | -0.090 |
| Tyrosol | -0.328 | -0.339 | -0.287 | -0.215 |
| Vanillic acid | -0.070 | 0.084 | 0.019 | -0.062 |
| Ligstroside derivative | -0.294 | -0.128 | -0.167 | -0.270 |
| p-cumaric acid | 0.092 | 0.282 | 0.213 | 0.111 |
| Hydroxydecarboxymethil elenolic acid | -0.126 | 0.036 | 0.004 | 0.006 |
| Elenolic acid | -0.155 | 0.050 | 0.009 | -0.077 |
| Dialdehydic for of oleuropein aglycon | 0.295 | 0.504** | 0.512** | 0.428* |
| Oleuropein aglycon (3,4-DHPEA) | 0.677*** | 0.881*** | 0.757*** | 0.705*** |
| Oleuropein aglycon | 0.256 | 0.208 | 0.199 | 0.385* |
| Lygstroside aglycon (p-HPEA-EA) | 0.709*** | 0.743*** | 0.669*** | 0.632*** |
| Oleuropein aglycon | 0.584** | 0.589** | 0.564** | 0.621*** |
| Luteolin | 0.089 | 0.165 | 0.143 | 0.169 |
| lygstroside aglycon (p-HPEA-EA) | 0.477** | 0.343** | 0.368** | 0.417** |
| Apigenin | 0.042 | 0.289 | 0.286 | 0.210 |
| Unknown 1 | 0.002 | 0.210 | 0.049 | 0.013 |
| Unknown 2 | -0.025 | -0.046 | -0.009 | -0.097 |
| Unknown 3 | 0.136 | -0.001 | -0.048 | 0.246 |

*p <0.05; **p <0.01; ***p <0.001.
